# Navigating practical challenges in the clinical deployment of AI for histopathological growth pattern classification

**DOI:** 10.1097/JS9.0000000000004724

**Published:** 2026-01-13

**Authors:** Haoxiang Qin, Zhihang Fan, Xiaoyu Yang, Bin Ma

**Affiliations:** Department of Colorectal Surgery, Cancer Hospital of China Medical University, Cancer Hospital of Dalian University of Technology, Liaoning Cancer Hospital & Institute, Shenyang, China

*To the Editor*,

We read with great interest the prospective study by Lin *et al* detailing the development and validation of COFFEE, an artificial intelligence (AI) model for classifying histopathological growth patterns (HGPs) in colorectal liver metastases (CRLM)[1]. The authors present compelling evidence of the model’s high diagnostic accuracy and its potential to augment pathologist performance, a significant stride toward addressing the critical shortage of specialized pathologists. However, as we consider the translation of such advanced AI tools from research to routine clinical practice, several practical and philosophical challenges emerge. These challenges, if unaddressed, may hinder the realization of AI’s full potential in precision oncology.

The positioning of AI as a collaborative “copilot,” however, immediately confronts a fundamental dilemma rooted in the “black box” nature of its decision making[2]. While such tools can achieve superior performance in specific tasks, this very strength complicates the human–AI partnership. Pathologists retain ultimate diagnostic responsibility, yet they are asked to rely on a system that provides high-confidence predictions without a transparent rationale. Attention heatmaps may show where the model looked, but they fail to explain why certain regions are decisive for classifying a desmoplastic versus a replacement pattern. This opacity creates a professional and ethical bind. It risks fostering uncritical overreliance that could erode diagnostic autonomy or, conversely, lead to rejection of the tool due to an inability to trust what one cannot understand[3]. Without progress toward interpretability that aligns with the pathologist’s feature-based reasoning, integrating such powerful but inscrutable algorithms into high-stakes clinical decision making remains profoundly challenging.

Furthermore, the model’s stellar performance is intrinsically linked to the data it was born from. The question of generalizability and embedded bias looms large. The COFFEE model was trained and validated on data from a single, high-caliber institution in China. CRLM biology, however, may exhibit geographic, ethnic, and environmental variations. More pragmatically, pre-analytical variables – tissue fixation protocols, staining techniques, and scanner models – vary widely across laboratories globally. An AI model can become a “digital expert” for the Sixth Affiliated Hospital of Sun Yat-sen University, but will it maintain its prowess when presented with slides from a community hospital with different protocols? Studies have shown that even slight variations in staining can dramatically degrade the performance of deep learning models in pathology[4]. The authors acknowledge the need for multicenter validation, which is not merely a procedural step but a necessity to uncover hidden biases and ensure equitable performance across diverse patient populations and healthcare settings[5].

Finally, the vision of widespread deployment collides with the realities of healthcare infrastructure. The computational demands of running a model like COFFEE, which involves processing gigapixel whole-slide images through complex Transformer architectures, are non-trivial. Does every pathology department have access to the necessary high-performance computing resources, secure data transfer pipelines, and IT support? The risk is creating a new digital divide, where AI-augmented precision diagnosis becomes the privilege of well-resourced academic centers, exacerbating existing disparities in cancer care[6]. The sustainability of such tools depends not only on algorithmic accuracy but on their integration into scalable, cost-effective, and user-friendly clinical workflows.

Lin *et al* have undoubtedly provided a powerful proof of concept. The COFFEE model represents a significant technical achievement. The true test, however, lies ahead. Moving from a successful prospective study to robust, trusted, and equitable clinical integration requires a parallel focus on the “soft” challenges: building appropriate trust and collaboration paradigms, demystifying the AI decision-making process, rigorously validating across the tapestry of global healthcare, and designing for real-world feasibility. The ultimate question is not whether AI can classify HGP patterns with high accuracy, but how we can thoughtfully shepherd this technology into the clinic to ensure it truly serves the complex, human-centric endeavor of patient care.

## Data Availability

No new data were generated or analyzed in this correspondence. Data sharing is not applicable to this article.
